# Machine learning-based identification of determinants of pulse pressure in pregnant women

**DOI:** 10.1016/j.gloepi.2026.100245

**Published:** 2026-01-07

**Authors:** Merga Abdissa Aga

**Affiliations:** Department of Statistics, Salale University, Fiche, Ethiopia

**Keywords:** Pulse pressure, Pregnancy, Machine learning, Random Forest, XGBoost, Generalized linear mixed model

## Abstract

**Background:**

Pulse pressure (PP) is an important marker of arterial stiffness and cardiovascular risk during pregnancy, yet its longitudinal determinants remain insufficiently characterized, particularly in low-resource settings.

**Objective:**

To identify determinants of longitudinal pulse pressure among pregnant women using machine learning approaches and to compare their predictive performance with a conventional mixed-effects modeling framework.

**Methods:**

We conducted a retrospective cohort study of 549 pregnant women attending public antenatal care services at Bishoftu General Hospital, Oromia region, Ethiopia, comprising 2760 repeated pulse pressure measurements. Pulse pressure was modeled as a continuous longitudinal outcome. Predictors included maternal sociodemographic characteristics, clinical measurements, obstetric history, and gestational age at each visit. A generalized linear mixed model, random forest regression, and XGBoost regression were applied. Participant-level data partitioning was used for model training and evaluation, and predictive performance was assessed using root mean squared error (RMSE) and mean absolute error (MAE).

**Results:**

Tree-based machine learning models showed improved predictive performance compared with the mixed-effects model, indicating the presence of nonlinear and time-dependent relationships between predictors and pulse pressure trajectories. Maternal age, body weight, gestational age, and pulse pressure values from previous visits consistently contributed to pulse pressure prediction.

**Conclusion:**

Machine learning methods applied to longitudinal antenatal data provide a flexible and effective framework for modeling pulse pressure dynamics during pregnancy. This approach enhances understanding of key clinical and temporal determinants and may support improved cardiovascular risk assessment in maternal health care settings.

## Introduction

Hypertensive disorders of pregnancy (HDP), including preeclampsia, gestational hypertension, and chronic hypertension, remain among the leading causes of maternal and perinatal morbidity and mortality worldwide, with the highest burden observed in low- and middle-income countries [[1], [2], [3]]. Vascular dysfunction during pregnancy not only affects immediate maternal outcomes but may also have long-term cardiovascular implications [4,5].

Pulse pressure (PP)—defined as the difference between systolic and diastolic blood pressure—is increasingly recognized as a surrogate marker of arterial stiffness and a predictor of cardiovascular risk [[6], [7], [8]]. In non-pregnant populations, elevated PP has been linked to left ventricular hypertrophy, atherosclerosis, and increased risk of cardiovascular events [9,10]. In pregnancy, however, longitudinal studies examining PP trends and determinants are notably scarce, particularly in low-resource settings where HDP are most prevalent [11].

Pregnancy induces substantial hemodynamic changes, including increased cardiac output and decreased systemic vascular resistance, which affect blood pressure components over time [12,13]. While most studies focus on absolute blood pressure values or dichotomous hypertension outcomes, few have modeled PP as a continuous longitudinal variable, capturing dynamic vascular changes throughout gestation. This represents a critical gap in maternal cardiovascular research, especially given the predictive utility of PP for adverse perinatal outcomes such as preterm birth, fetal growth restriction, and placental abruption [[14], [15], [16]].

Furthermore, much of the current literature employs cross-sectional designs, which fail to account for intra-individual variations and evolving risk profiles. Longitudinal analytical approaches, incorporating repeated PP measurements, provide a more comprehensive understanding of maternal hemodynamics and may enable earlier identification of high-risk groups [17,18].

Several maternal factors have been associated with abnormal blood pressure trajectories [3,19,20]. However, the predictive capacity and relative importance of these factors on PP patterns across pregnancy remain underexplored.

Traditional statistical models may have limited ability to capture complex, nonlinear interactions between multiple predictors. Machine learning (ML) approaches, including tree-based algorithms and ensemble methods, can address these limitations by flexibly modeling high-dimensional relationships without strict parametric assumptions, thereby improving prediction accuracy and variable importance assessment [[21], [22], [23]].

This study addresses an important methodological and applied gap in maternal cardiovascular research by modeling pulse pressure as a longitudinal outcome using machine learning approaches. Leveraging repeated antenatal measurements collected across gestation, we incorporate historical pulse pressure values from prior visits to account explicitly for temporal dependence. We further provide a direct comparison between tree-based machine learning models Random Forest and Extreme Gradient Boosting (XGBoost) and a conventional generalized linear mixed-effects model (GLMM) within the same data structure. By integrating nonlinear modeling with routinely collected clinical and obstetric variables, this work offers a flexible framework for identifying determinants of pulse pressure trajectories during pregnancy, with particular relevance for low-resource health care settings.

## Methods

### Study design and setting

This study employed a retrospective longitudinal cohort design utilizing routinely collected clinical data from pregnant women attending Bishoftu General Hospital, located in Bishoftu town, Ethiopia. The hospital provides comprehensive antenatal care (ANC), delivery services, and postnatal follow-up to a diverse patient population. All eligible ANC visits occurring between January 2023 and December 2023 were considered for inclusion.

From a practical clinical perspective, the dataset reflects routine antenatal care (ANC) follow-up in a public hospital setting. Pulse pressure measurements were obtained during scheduled ANC visits as part of standard blood pressure assessment, requiring no additional procedures beyond usual care. The repeated measurements across pregnancy capture real-world physiological changes rather than experimental conditions.

The predominance of low-risk pregnancies mirrors typical ANC populations in similar low-resource settings, making the data representative of routine maternal health services. Importantly, the longitudinal structure allows identification of women whose pulse pressure trajectories deviate from expected patterns over time, which is clinically relevant for early detection of cardiovascular and hypertensive complications.

### Study population

Eligible participants included pregnant women who had at least two recorded blood pressure measurements during ANC visits, allowing for longitudinal analysis. Women with pre-existing chronic hypertension, missing outcome measurements, or incomplete data on key predictors were excluded. After applying these criteria, the final analytic sample consisted of 549 women, contributing a total of 2760 repeated blood pressure measurements.

### Method of data collection

Data were retrospectively extracted from medical records of pregnant women attending ANC at Bishoftu General Hospital between January 2023 and December 2023. Blood pressure measurements and relevant sociodemographic, obstetric, clinical, and family history variables were recorded by trained healthcare professionals as part of routine care. A blank copy of the data collection instrument used to extract antenatal and clinical information is provided as Supplementary Material S1. Regular data quality checks were conducted to identify inconsistencies, out-of-range values, and missing information prior to analysis.

### Outcome variable

The primary outcome is pulse pressure (PP), calculated as the difference between systolic and diastolic blood pressure:

$${\mathrm{PP}}_{\mathrm{ij}}={\mathrm{SBP}}_{\mathrm{ij}}-{\mathrm{DBP}}_{\mathrm{ij}}.$$

where ${\mathrm{PP}}_{\mathrm{ij}}\;$ represents the pulse pressure of the i^th^ woman at the j^th^ visit. Pulse pressure was analyzed as a continuous outcome across repeated visits.

#### Predictor's variables

Predictors were selected based on clinical relevance and prior literature and included maternal demographic, obstetric, clinical, and familial characteristics. These variables were grouped as follows:

**Table:** 

Category	Predictors
Demographics	Age, weight
Obstetric history	Gravidity(primigravida, multigravida), history of abortion (yes, no), history of preeclampsia(yes, no)
Pregnancy status	Gestational age (weeks), multiplicity (twins, single), ANC visits (<4, ≥4)
Clinical variables	Comorbidities (yes, no), complications developed (yes, no), anti-hypertension medication (yes, no)
Family history	Family history of BP (yes, no)

### Data curation and preprocessing

Prior to model development, the dataset underwent structured curation. Initially, all ANC records within the study period were screened. Records were excluded for chronic hypertension, fewer than two blood pressure measurements, or missing outcome data, resulting in a final dataset of 549 women with 2760 pulse pressure observations.

Continuous variables were assessed for plausibility using clinically defined ranges, and extreme values were reviewed against source records where available. No automated outlier removal was performed to preserve clinically meaningful variability. Missing values were imputed using median imputation for continuous variables, minimizing data loss while reducing potential bias. Categorical variables were encoded to ensure compatibility with machine learning algorithms. To capture temporal dependencies inherent in longitudinal data, lagged pulse pressure variables were constructed using measurements from previous ANC visits [24,25].

### Feature selection strategy

Predictor selection was based on clinical relevance and prior empirical evidence rather than data-driven variable screening. No univariate filtering, stepwise selection, or regularization-based exclusion was applied prior to model fitting. This strategy was adopted to enhance interpretability and reduce overfitting risk in the context of a moderate sample size. For machine learning models, all selected predictors were retained, and variable importance was assessed post hoc using model-specific importance measures.

### Temporal feature construction

To capture temporal dependence in longitudinal pulse pressure measurements, lagged pulse pressure values from the immediately preceding antenatal visit were included as time-dependent predictors. For each individual i at visit j, pulse pressure measured at visit j − 1 was incorporated as a covariate. This approach accounts for within-subject autocorrelation and short-term physiological dynamics without relying on model-derived residuals.

All lagged features were generated using observed clinical measurements only. During data partitioning, participant-level splitting ensured that all repeated measurements from an individual were confined to either the training or testing set, thereby preventing information leakage.

### Data volume and longitudinal structure

The final dataset comprised 2760 pulse pressure measurements obtained from 549 pregnant women, with a median of five ANC visits per woman (interquartile range: 2–8 visits). This longitudinal structure provided repeated within-subject observations over gestation, enabling the modeling of temporal dependencies in pulse pressure while preserving independence between subjects during training and testing. The available sample size and observation frequency were sufficient to support machine learning model development, subject-wise cross-validation, and robust out-of-sample performance evaluation.

### Training–testing split and validation strategy

The dataset was divided into training and testing sets using a subject-wise split to preserve the longitudinal structure and prevent information leakage [26]. All observations from a given woman were assigned exclusively to either the training or the testing set. A hold-out test set comprising approximately 20% of participants was reserved for final model evaluation, while the remaining 80% were used for model training and internal validation.

Within the training set, five-fold subject-wise cross-validation was employed for hyperparameter tuning and model selection. Model performance was subsequently evaluated on the independent hold-out test set to assess out-of-sample predictive accuracy. Because pulse pressure was modeled as a continuous outcome, model performance was quantified using regression-based metrics, including Root Mean Squared Error (RMSE), Mean Absolute Error (MAE), and the coefficient of determination (R^2^).

### Statistical models

#### Linear Mixed-Effects Model (LME)

As a classical baseline approach, we fitted a linear mixed-effects model to account for repeated measurements within individuals [27]. The model is specified as:

$${\mathrm{PP}}_{\mathrm{ij}}=\beta_{0}+X_{\mathrm{ij}}\beta +b_{i}+E_{\mathrm{ij}}.$$

where ${\mathrm{PP}}_{\mathrm{ij}}$is the pulse pressure for individual i at time j

$\beta_{0}$is the overall intercept

$X_{\mathrm{ij}}$is the vector of predictors for individual i at time j

βis the vector of fixed-effect coefficients

$b_{i}∼N\left(0,\sigma_{b}^{2}\right)$ is the random intercept accounting for between-subject variability

$E_{\mathrm{ij}}∼N\left(0,\sigma^{2}\right)$ is the residual error.

This model captures individual-specific baseline differences in PP and adjusts for measured covariates. Before fitting the LME, assumptions such as normality and homoscedasticity of residuals were evaluated [28]. Due to violations of these assumptions—specifically non-normal residuals and heteroscedasticity—we extended the analysis using a Generalized Linear Mixed-Effects Model (GLMM) [29].

Prior to fitting the LMM, we assessed model assumptions including normality and homoscedasticity of residuals. Due to violation of these assumptions (e.g., non-normal residuals, heteroscedasticity), we extended the analysis using a Generalized Linear Mixed-Effects Model (GLMM), which allows for non-normal error distributions and link functions better suited for the data distribution. The GLMM allows for non-normal error distributions and flexible link functions, making it better suited to the data distribution observed [29]. The model is expressed as:

$$g\left(\mu_{\mathrm{ij}}\right)=\beta_{0}+X_{\mathrm{ij}}\beta +b_{i}.$$

$\mu_{\mathrm{ij}}=E\left({\mathrm{PP}}_{\mathrm{ij}}/b_{i}\right)$ is the conditional mean of pulse pressure given the random effect,

$g\left(.\right)$ is the link function relating the mean to the linear predictor,

$b_{i}∼N\left(0,\sigma_{b}^{2}\right)$ is the random intercept accounting for between-subject variability.

For the pulse pressure data, we selected a family of gamma distribution with a log link function, accommodating the positive continuous nature and skewness of the outcome.

#### Random Forest (RF)

We implemented a Random Forest regressor adapted for longitudinal data by incorporating lagged pulse pressure and temporal covariates [30]. This ensemble method builds multiple regression trees to minimize prediction error without requiring assumptions of linearity or independence among predictors.

#### Extreme gradient boosting (XGBoost)

XGBoost models were trained using the same predictors and lagged variables as the Random Forest [31]. The model iteratively fits decision trees to residual errors, optimizing a regularized objective function to reduce overfitting. Hyperparameters—including learning rate, maximum tree depth, number of estimators, and subsampling rate—were tuned via grid search combined with 5-fold subject-wise cross-validation [32].

### Model parameters and selection criteria

Model parameters for all analytical approaches were selected based on established methodological recommendations and empirical tuning within the training data. For the generalized linear mixed-effects model (GLMM), fixed-effect coefficients were estimated via maximum likelihood, with a random intercept specified at the individual level to account for within-subject correlation arising from repeated antenatal measurements.

For machine learning models, hyperparameters were determined using grid search combined with cross-validation within the training set. For the random forest regression model, the number of trees, maximum tree depth, and minimum node size were tuned to balance predictive accuracy and model stability. For the Extreme Gradient Boosting (XGBoost) model, key hyperparameters—including learning rate, maximum tree depth, subsampling ratio, and number of boosting iterations—were optimized to minimize prediction error while preventing overfitting.

All parameter choices were constrained to ensure compatibility with the longitudinal structure of the data and clinical plausibility of pulse pressure trajectories. Model specifications were validated by assessing prediction stability and consistency across cross-validation folds, thereby ensuring that selected parameters adequately represented the underlying pulse pressure dynamics observed in the study population.

### Generalized modeling algorithm

This study followed a structured analytical workflow to model longitudinal pulse pressure dynamics and identify key determinants using both conventional and machine learning approaches.

*Step 1: Data Preparation*

Raw antenatal care records were screened for implausible physiological values and duplicate observations. The dataset was organized in long format, with repeated pulse pressure measurements indexed by participant and antenatal visit. Pulse pressure was calculated as the difference between systolic and diastolic blood pressure. Time-dependent features were constructed, including lagged pulse pressure values from prior visits.

*Step 2: Data Partitioning*

To preserve the longitudinal structure of the data and prevent information leakage, the dataset was partitioned at the participant level into training and testing subsets. All repeated measurements from a given participant were assigned exclusively to a single subset.

*Step 3: Model Specification*

A generalized linear mixed-effects model was specified with participant-level random effects to account for within-subject correlation arising from repeated measurements. In parallel, machine learning regression models—Random Forest and Extreme Gradient Boosting (XGBoost)—were specified using maternal sociodemographic, clinical, obstetric, and temporal predictors.

*Step 4: Model Training and Tuning*

Parameters of the mixed-effects model were estimated using maximum likelihood methods. Hyperparameters for the machine learning models were optimized within the training data using cross-validation. Model configurations were selected to balance predictive accuracy and stability while minimizing overfitting.

*Step 5: Model Evaluation*

Trained models were applied to the held-out test dataset. Predictive performance was evaluated using root mean squared error and mean absolute error. Model performance was compared across analytical approaches to assess relative predictive capability.

*Step 6: Interpretation and Robustness Assessment*

Variable importance measures were extracted from machine learning models to identify influential determinants of pulse pressure. Sensitivity analyses were conducted to evaluate the robustness of results to alternative parameter choices and model specifications.

*Output*

Predicted longitudinal pulse pressure trajectories, ranked determinants of pulse pressure, and comparative performance metrics across models.

This algorithm reflects the longitudinal structure of antenatal pulse pressure data by explicitly accounting for repeated measurements and temporal dependence. Participant-level data partitioning ensures valid model evaluation without information leakage. The combined use of mixed-effects modeling and machine learning regression enables both interpretable inference and flexible nonlinear prediction, making the approach well aligned with the study data, analytical objectives, and clinical context of antenatal care.

**Fig. 1:**
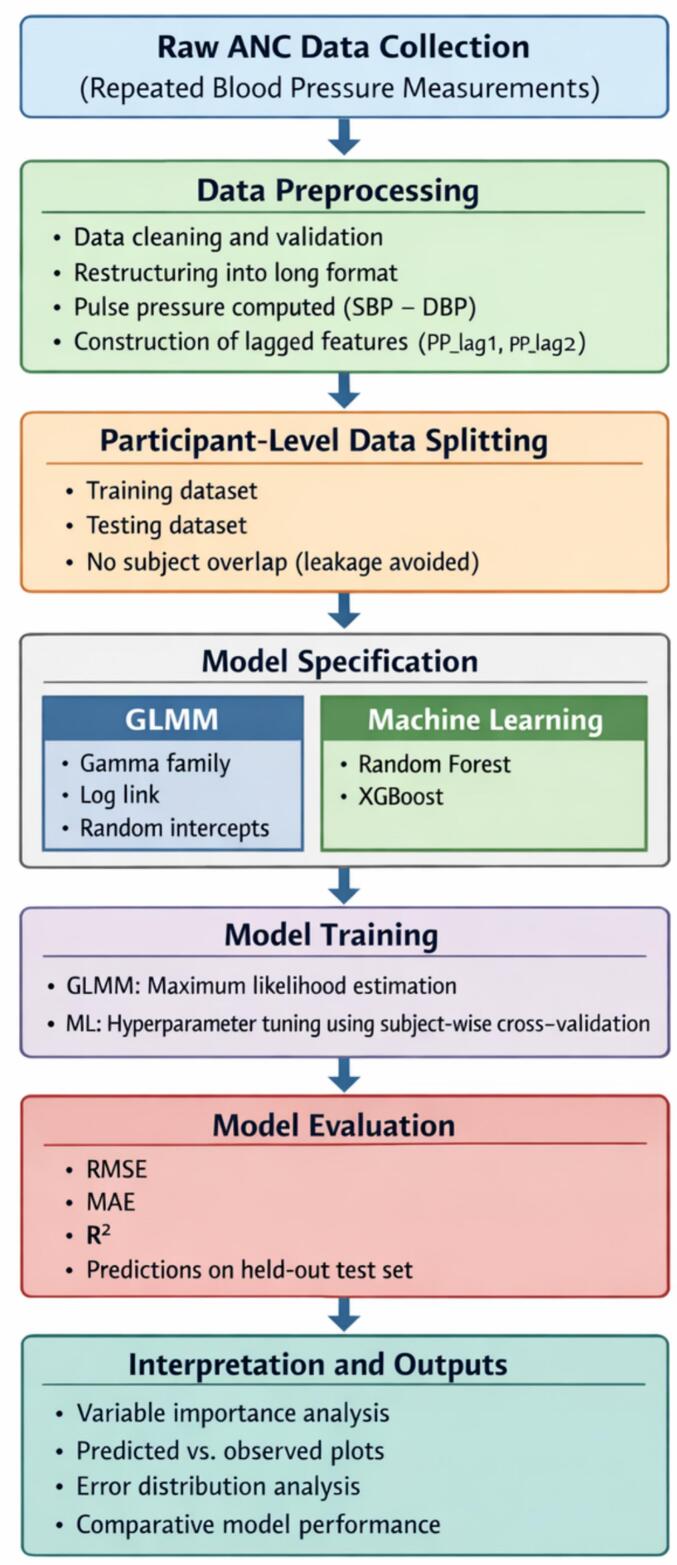
Analytical workflow for modeling longitudinal pulse pressure in pregnant women.

The flowchart summarizes the complete analytical pipeline, starting from raw antenatal care (ANC) data collection with repeated blood pressure measurements, followed by data preprocessing and construction of time-dependent features. Participant-level data splitting was applied to preserve the longitudinal structure and avoid information leakage. Model specification included a generalized linear mixed model (GLMM) with a Gamma distribution and log link, as well as machine learning models (Random Forest and XGBoost). Models were trained and evaluated using RMSE, MAE, and R^2^ on a held-out test set. Final outputs include variable importance measures, prediction accuracy assessments, and comparative model performance (Fig. 1).

### Sensitivity analysis of model parameters

A sensitivity analysis was conducted to assess the robustness of model behavior with respect to key parameter choices and to evaluate how variations in these parameters influence pulse pressure predictions. This analysis strengthens internal validity by demonstrating that the study findings are not driven by arbitrary modeling assumptions.

For the generalized linear mixed-effects model (GLMM), sensitivity was examined by varying distributional assumptions and temporal structure. Specifically, alternative link functions (identity and log links) and variance specifications were evaluated, and models were re-estimated using different lag structures (lag 1 only and lags 1–3). The direction and relative magnitude of fixed-effect estimates remained stable across model specifications, and predictive performance metrics showed minimal variation, indicating robustness to reasonable changes in model structure.

For the machine learning models, sensitivity analysis focused on key hyperparameters. In the random forest model, the number of trees, maximum tree depth, and minimum node size were varied within commonly recommended ranges. Predictive performance metrics (root mean squared error [RMSE], mean absolute error [MAE], and explained variance [R^2^]) remained consistent across these settings, with the random forest model maintaining superior predictive accuracy. In the XGBoost model, sensitivity to the learning rate, tree depth, and subsampling ratio was evaluated using participant-level cross-validation. Model performance exhibited gradual degradation outside optimal ranges, indicating stable learning behavior without evidence of overfitting.

In addition, the influence of temporal features was examined by refitting models after removing second-order lagged pulse pressure terms. Although predictive accuracy decreased slightly, the relative ranking of model performance and the importance of key demographic and clinical predictors remained unchanged, confirming the stability of the main findings.

Overall, the sensitivity analyses demonstrate that the study results are robust to reasonable variations in model parameters and specifications, supporting the reliability of the estimated associations and the ability of the models to capture the underlying longitudinal dynamics of pulse pressure during pregnancy.

### Numerical model fitting and training procedure

Numerical model fitting was conducted to characterize how each analytical approach captured longitudinal pulse pressure dynamics and to ensure methodological comparability across models. All models were trained using the same subject-wise training dataset to preserve within-participant dependence structures.

For the generalized linear mixed model (GLMM), numerical estimation was performed using maximum likelihood methods. Participant-level random intercepts were included to account for within-subject correlation arising from repeated antenatal visits. Model convergence was assessed using standard diagnostic criteria, including stability of parameter estimates and variance components.

For the machine learning models, Random Forest and Extreme Gradient Boosting (XGBoost), numerical fitting involved iterative optimization procedures. Hyperparameters were tuned exclusively within the training dataset using subject-wise cross-validation to minimize prediction error while avoiding information leakage. Final model configurations were selected based on stable convergence behavior and consistent predictive performance across validation folds.

All numerical fitting procedures were implemented using established statistical and machine learning libraries, and identical training data partitions were applied across models to ensure fair comparison.

### Model evaluation

Predictive performance was evaluated on the held-out test set using Root Mean Squared Error (RMSE), Mean Absolute Error (MAE), and the Coefficient of Determination (R^2^) on held-out test sets. Subject-wise k-fold cross-validation was employed to preserve the longitudinal data structure, with performance metrics averaged across folds to ensure a fair comparison among models [33].

## Results

The Results section is organized as follows. First, descriptive characteristics of the study population and pulse pressure measurements are summarized. Second, longitudinal patterns of pulse pressure are illustrated graphically. Third, results from the generalized linear mixed model are presented. Finally, predictive performance and variable importance results from machine learning models are reported.

### Descriptive characteristics of the study population

A total of 2760 pulse pressure (PP) measurements were obtained from 549 pregnant women with repeated antenatal visits. Summary statistics for continuous variables are presented in Table 1. The mean pulse pressure was 45.59 mmHg (SD = 9.52), indicating moderate variability across observations. The mean maternal age was 27.68 years (SD = 5.44), and the average maternal weight was 69.12 kg (SD = 9.39). The mean gestational age at measurement was 38.81 weeks (SD = 2.32), reflecting that most observations were collected in late pregnancy.

**Table 1:** Summary of covariates.

Variable	Mean	SD
Pulse Pressure	45.59	9.52
Age in years	27.68	5.44
Weight in kg	69.12	9.39
GA in weeks	38.81	2.32

Categorical characteristics of the study population are summarized in Table 2. The majority of participants reported no family history of hypertension (91.8%), no history of preeclampsia (93.3%), and no previous abortions (93.6%). Most women were multigravida (61.4%) and attended at least four antenatal care visits (98.4%). Singleton pregnancies accounted for 97.6% of observations.

**Table 2:** Summary of predictor variables.

Variable	Category	Frequency	Percent
Family history BP	No	504	91.8%
	Yes	45	8.2%
History preeclampsia	No	512	93.3%
	Yes	37	6.7%
Previous abortion	No	514	93.6%
	Yes	35	6.4%
Gravidity	primigravida	212	38.6%
	Multigravida	337	61.4%
Number ANC	<4	9	1.6%
	≥4	540	98.4%
Pregnant Multiplicity	Singleton	536	97.6%
	Twin	13	2.4%
Any comorbidity	No	529	96.4%
	Yes	20	3.6%
If, yes	Diabetes Mellitus	9	45%
	Renal disease	4	20%
	History of hypertension	6	30%
	Others	2	10%
Antihypertensive	No	478	87.1%
	Yes	71	12.9%
Develop complications	No	503	91.6%
	Yes	46	8.4%
If yes,	HELLP Syndrome	37	80.4%
	Other	9	19.6%

Comorbid conditions were uncommon, with only 3.6% of participants reporting at least one chronic condition. Among these, diabetes mellitus was the most frequently reported (45.0%), followed by a history of hypertension (30.0%) and renal disease (20.0%). The majority of women were not receiving antihypertensive treatment during pregnancy (87.1%). Pregnancy-related complications were observed in 8.4% of participants, with HELLP syndrome accounting for 80.4% of reported complications.

**Fig. 2:**
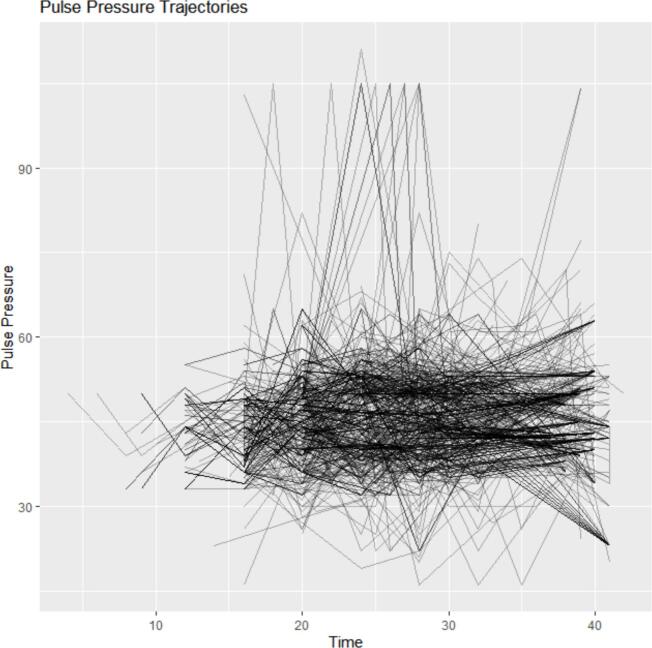
Spaghetti plot of pulse pressure over time.

The spaghetti plot demonstrates substantial variability in pulse pressure trajectories both within and between individuals over time. While some participants exhibit relatively stable pulse pressure across antenatal visits, others show pronounced fluctuations, indicating heterogeneity in longitudinal patterns. This variability is evident across gestational ages and highlights the presence of subject-specific temporal dynamics in pulse pressure measurements (Fig. 2).

Given the longitudinal nature of the pulse pressure measurements collected at multiple time points for each participant, lagged values of pulse pressure were incorporated as predictors in the generalized linear mixed model (GLMM). This design accounts for temporal dependence within individuals by allowing current pulse pressure to depend on measurements from previous visits.

In the GLMM and machine learning models, lagged pulse pressure values from the previous visit (lag 1) and, where appropriate, the second previous visit (lag 2) were included as predictors. These features captured short-term temporal dependence, while additional lags did not improve predictive performance and were excluded.

A Gamma distribution with a log link was employed to accommodate the positively skewed distribution of pulse pressure, while adjusting for relevant demographic and clinical covariates.

Table 3 presents the fixed and random effects estimates from the generalized linear mixed model. Lagged pulse pressure at the previous visit showed a positive association with current pulse pressure, whereas the second lag exhibited a weaker association. Time, maternal age, weight, and gestational age were positively associated with pulse pressure.

**Table 3:** Fixed and random effects estimates from the GLMM on pulse pressure.

Covariate	Estimate	Std. Error	z value	*p*-value
Fixed Effects				
(Intercept)	4.0425	0.0996	40.57	<0.0001
Lag1	0.05	0.0132	3.78	<0.01
Lag2	0.03	0.0240	1.25	0.08
Time	0.00105	0.00047	2.24	0.02
Age	0.00301	0.00092	3.29	0.001
Weight	0.00213	0.00048	4.43	<0.0001
GA in weeks	0.00408	0.00212	1.92	0.005
Family history BP	−0.01358	0.01777	−0.76	0.4445
Gravidity	−0.01143	0.01041	−1.10	0.2722
History preeclampsia	−0.0772	0.02029	−3.8	0.004
Previous abortion	0.03728	0.02081	1.79	0.07
Number ANC	−0.01904	0.00538	−5.38	0.01
Pregnant Multiplicity	−0.09111	0.02433	−3.75	0.0002
Any comorbidity	0.06310	0.02333	2.70	0.007
Antihypertensive	−0.03618	0.01589	−2.28	0.0228
Develop complications	0.01231	0.01777	0.69	0.4886
Random Effect	Variance	Standard Deviation		
ID (Intercept)	0.0944	0.031		
Residuals	0.914			

A history of preeclampsia and antihypertensive treatment were associated with lower pulse pressure. Women attending four or more antenatal care visits and those with singleton pregnancies exhibited lower pulse pressure compared with their counterparts. The presence of comorbidity was associated with higher pulse pressure. Other covariates, including family history of hypertension, gravidity, previous abortion, and development of complications, showed no meaningful association with pulse pressure.

Table 4 summarizes the predictive performance of the generalized linear mixed model (GLMM), Random Forest, and XGBoost models evaluated on the held-out test dataset. The GLMM exhibited the highest prediction error, with a mean RMSE of 9.46 (SD = 1.56), MAE of 3.78, and an R^2^ of 0.81.

**Table 4:** Comparative Cross-Validation Metrics of Pulse Pressure Prediction Models.

Model	Mean RMSE	SD RMSE	Mean MAE	R^2^
GLMM	9.464	1.56	3.78	0.81
Random Forest	2.74	0.52	1.57	0.92
XGBoost	3.41	0.80	1.74	0.88

In contrast, machine learning models achieved substantially lower prediction errors. The Random Forest model demonstrated the best performance, with a mean RMSE of 2.74 (SD = 0.52), MAE of 1.57, and R^2^ of 0.92. The XGBoost model also performed well, yielding a mean RMSE of 3.41 (SD = 0.80), MAE of 1.74, and R^2^ of 0.88.

Across all evaluation metrics, tree-based machine learning models outperformed the mixed-effects model in predicting longitudinal pulse pressure measurements.

The superior predictive accuracy of Random Forest and XGBoost compared with GLMM suggests that machine learning models may provide more reliable tools for monitoring maternal cardiovascular dynamics during pregnancy. Such improvements in prediction could support early identification of women at risk for hypertensive complications in routine antenatal care settings.

**Fig. 3:**
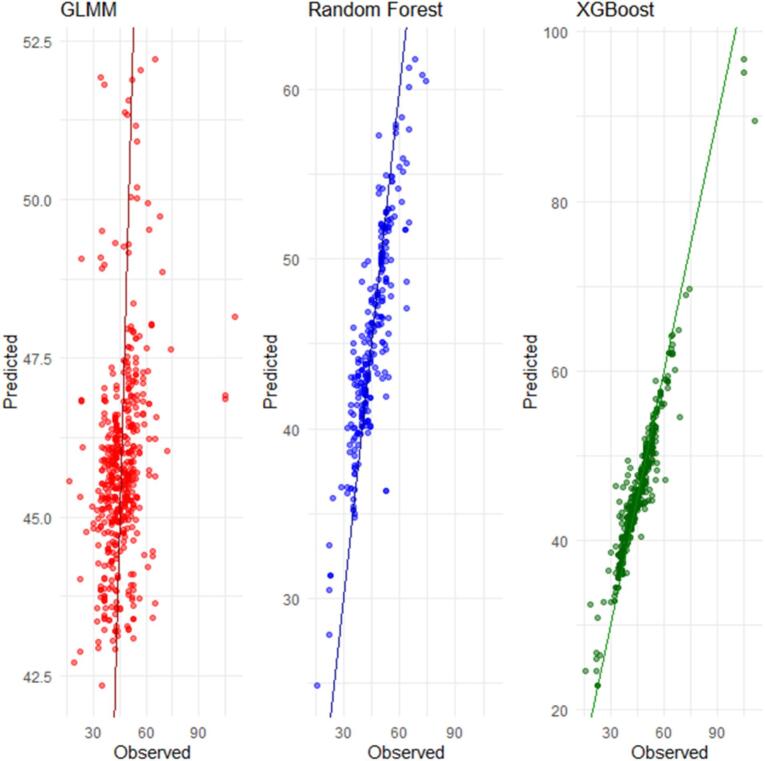
Comparison of predicted and observed pulse pressure across models.

The predicted versus observed pulse pressure plots demonstrate that the Random Forest and XGBoost models closely track the observed values across the test dataset, with predictions concentrated near the identity line. In contrast, predictions from the generalized linear mixed model show greater dispersion, indicating reduced agreement with observed pulse pressure values (Fig. 3).

**Fig. 4:**
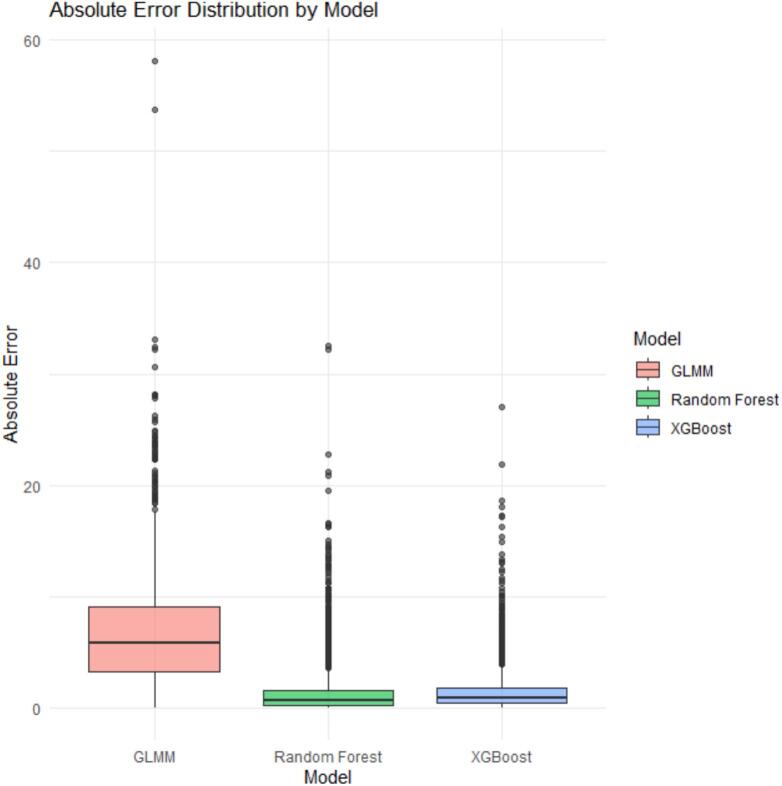
Boxplot of absolute prediction errors for GLMM, Random Forest, and XGBoost models*.*

The distribution of absolute prediction errors further illustrates differences in model performance (Fig. 4). The Random Forest model exhibits the lowest median absolute error and the narrowest interquartile range, reflecting both higher accuracy and greater consistency. XGBoost shows comparable performance, with slightly higher variability, while the GLMM displays larger and more dispersed errors. These graphical results are consistent with the numerical performance metrics reported in Table 4.

**Fig. 5:**
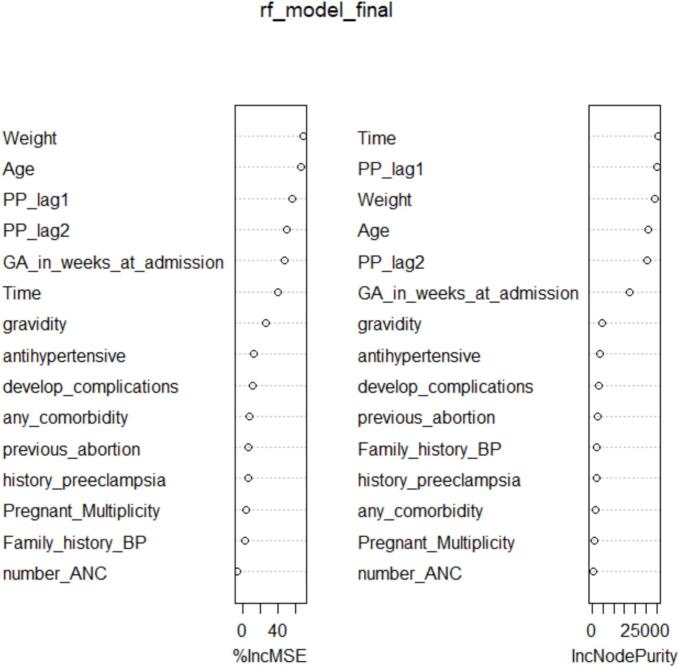
Random forest variable importance for predicting pulse pressure.

Fig. 5 presents the variable importance measures from the Random Forest model used to predict pulse pressure. Predictor importance was assessed using permutation-based percentage increase in mean squared error (%IncMSE) and node purity. Lagged pulse pressure values (PP_lag1 and PP_lag2), maternal weight, and age ranked highest across both importance measures, indicating their strong contribution to predictive performance.

Table 5 summarizes the Random Forest model performance and corresponding importance metrics for all predictors. Variables related to prior pulse pressure history and maternal physiological characteristics consistently ranked above obstetric and clinical history variables. Gestational age at admission and time also contributed meaningfully to prediction, while variables such as family history of blood pressure, gravidity, and antenatal care attendance showed relatively lower importance.

**Table 5:** Summary of random forest model performance and variable importance.

Variable	%IncMSE (Higher = More Important)	IncNodePurity (Higher = More Important)
Weight	69.67	28,958.36
Age	66.81	25,909.85
PP_lag1	56.21	29,706.58
PP_lag2	50.25	25,509.62
GA_in_weeks_at_admission	47.32	17,255.48
Time	40.44	30,254.74
gravidity	26.74	4669.41
antihypertensive	12.77	3640.54
develop_complications	11.41	3413.26
any_comorbidity	7.83	1871.53
previous_abortion	7.00	2462.24
history_preeclampsia	6.91	2162.63
Family_history_BP	2.99	2240.98
Pregnant_Multiplicity	3.92	1182.40
number_ANC	3.74	923.05

Overall, these results indicate that temporal dependence and maternal physiological characteristics are key contributors to accurate pulse pressure prediction in the Random Forest model.

The Random Forest model identified several key predictors of pulse pressure among the candidate variables. Notably, *Weight and Age* emerged as the most influential factors, exhibiting the highest percentage increase in mean squared error (%IncMSE) when permuted, with values of approximately 69.7% and 66.8%, respectively. This indicates that these variables contribute substantially to the model's predictive accuracy. Additionally, the inclusion of lagged pulse pressure values (*PP_lag1* and *PP_lag2)* demonstrated significant importance (%IncMSE of 56.2% and 50.2%), highlighting the strong temporal dependency inherent in the longitudinal pulse pressure measurements. Other notable predictors included gestational age at admission and time, suggesting physiological and temporal effects on pulse pressure dynamics. Overall, the importance metrics suggest that both demographic factors and historical pulse pressure readings are critical in accurately modeling pulse pressure variation in pregnant women (Table 5).

Fig. 5 plot displays the relative importance of predictor variables in the XGBoost model for pulse pressure prediction. Importance is measured by the contribution of each variable to the model's predictive performance, highlighting key features driving the outcome.

**Fig. 6:**
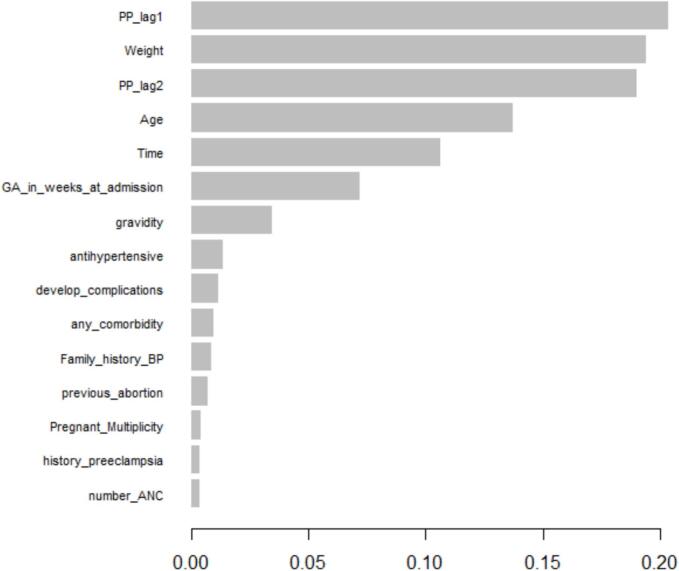
Variable importance plot from XGBoost model.

Fig. 6 displays the relative importance of predictor variables in the XGBoost model for pulse pressure prediction. Feature importance was primarily assessed using Gain, which reflects each variable's contribution to improving model accuracy, with supporting information from Cover and Frequency.

Table 6 summarizes the importance metrics for all predictors. Lagged pulse pressure values (PP_lag1 and PP_lag2) ranked highest, followed by maternal weight and age, indicating that recent pulse pressure history and maternal physiological characteristics were the strongest contributors to model performance. Temporal variables, including time and gestational age at admission, also contributed meaningfully, while obstetric and clinical history variables showed comparatively lower importance.

**Table 6:** Summary of variable importance in the XGBoost model.

Feature	Gain	Cover	Frequency	Importance (Gain)
PP_lag1	0.2036	0.1955	0.1810	0.2036
Weight	0.1941	0.1587	0.1437	0.1941
PP_lag2	0.1898	0.1527	0.1388	0.1898
Age	0.1374	0.1552	0.1542	0.1374
Time	0.1062	0.0958	0.1724	0.1062
GA_in_weeks_at_admission	0.0721	0.1106	0.0856	0.0721
Gravidity	0.0348	0.0159	0.0234	0.0348
Antihypertensive	0.0136	0.0196	0.0194	0.0136
Develop_complications	0.0115	0.0263	0.0187	0.0115
Any_comorbidity	0.0097	0.0193	0.0115	0.0097
Family_history_BP	0.0084	0.0063	0.0137	0.0084
Previous_abortion	0.0072	0.0103	0.0124	0.0072
Pregnant_Multiplicity	0.0041	0.0120	0.0077	0.0041
History_preeclampsia	0.0039	0.0128	0.0114	0.0039
Number _ANC	0.0031	0.0157	0.0105	0,0031

Overall, the XGBoost results are consistent with those from the Random Forest model, emphasizing the importance of temporal dependence and maternal characteristics in predicting longitudinal pulse pressure.

The identification of maternal weight, age, and prior pulse pressure values as dominant predictors highlights clinically accessible factors that can be leveraged in antenatal clinics without additional diagnostic burden. These findings underscore the potential for integrating machine learning–based risk stratification into existing maternal health workflows.

## Discussion

This study aimed to model longitudinal pulse pressure trajectories in pregnant women using both traditional statistical and advanced machine learning methods, with the objectives of identifying key predictors and enhancing prediction accuracy. Our findings reveal that machine learning models—specifically Random Forest and XGBoost—significantly outperform the Generalized Linear Mixed Model (GLMM) in terms of predictive accuracy and variance explained.

While the GLMM appropriately accounts for repeated measures and individual-level random effects, it showed limited predictive capability, as indicated by relatively high root mean squared error (RMSE) and low coefficient of determination (R^2^). This limitation likely stems from the linearity and additive assumptions intrinsic to GLMMs, which may fail to capture the complex nonlinear relationships and temporal dependencies inherent in longitudinal pulse pressure data. Such observations are consistent with previous cardiovascular biomarker studies highlighting challenges in modeling dynamic physiological processes using traditional parametric methods [34,35].

In contrast, Random Forest and XGBoost—both ensemble-based machine learning algorithms—effectively modeled nonlinear effects and complex interactions among predictors. These models achieved substantially improved performance metrics, demonstrating their robustness in handling high-dimensional clinical data with temporal structure [30,31]. Our results align with prior research endorsing tree-based machine learning approaches as superior tools for analyzing longitudinal biomedical data [36,37].

Variable importance analyses consistently identified lagged pulse pressure measurements (PP_lag1 and PP_lag2) as the most influential predictors, underscoring the strong temporal autocorrelation characteristic of longitudinal pulse pressure profiles. This finding corroborates earlier work emphasizing the critical role of prior blood pressure values in forecasting hypertensive disorders during pregnancy [38,39]. Demographic variables such as maternal weight and age also emerged as substantial contributors, consistent with known physiological changes affecting vascular compliance and hemodynamics during gestation [40].

Interestingly, clinical factors including antihypertensive treatment, history of preeclampsia, and comorbidities had relatively lower importance in the machine learning models compared to traditional models. This pattern may reflect complex, nonlinear interactions and variable prevalence that influence model-driven importance differently than hypothesis-driven parametric approaches. Such discrepancies echo prior findings where machine learning can down-weight clinically intuitive predictors if their predictive value in the dataset is limited [41].

These results support the growing consensus that machine learning techniques present promising alternatives or complementary tools to traditional statistical modeling in medical research, particularly for capturing intricate, nonlinear longitudinal data patterns [42,43]. Their flexibility and fewer parametric assumptions facilitate detection of subtle physiological signals vital for early risk stratification and personalized clinical management in pregnancy.

Nonetheless, challenges remain regarding the interpretability of machine learning models, which can hinder clinical adoption [44,45]. Future work should focus on integrating explainability frameworks such as SHAP (SHapley Additive explanations) and LIME (Local Interpretable Model-agnostic Explanations), as well as external validation of models in diverse populations, to enhance trust and facilitate translation of predictive insights into clinical decision-making [46].

From a health system perspective, the findings demonstrate that routinely collected antenatal data can be leveraged to model pulse pressure trajectories without additional clinical burden. The ability of machine learning models to accurately predict pulse pressure using prior measurements and basic maternal characteristics suggests potential application in early warning systems within antenatal clinics.

Identifying women whose pulse pressure deviates from expected longitudinal patterns may support targeted monitoring, timely referral, and preventive interventions, particularly in low-resource settings where advanced diagnostic tools are limited. The approach aligns with scalable, data-driven decision support strategies in maternal health care.

Compared with traditional mixed-effects models commonly used in longitudinal maternal health research, the machine learning approaches in this study demonstrated substantially improved predictive accuracy. Prior studies modeling blood pressure outcomes during pregnancy have largely relied on parametric regression frameworks, which may struggle to capture nonlinear and time-dependent relationships.

The improved performance of Random Forest and XGBoost observed here is consistent with recent applications of machine learning in obstetric risk prediction, suggesting that flexible, data-adaptive models may offer advantages over conventional statistical techniques when sufficient longitudinal data are available.

In conclusion, this study demonstrates that Random Forest and XGBoost provide superior predictive performance over GLMM for modeling longitudinal pulse pressure in pregnant women. Among the machine learning methods, Random Forest notably outperformed XGBoost, delivering higher accuracy and more consistent predictions. Leveraging machine learning—especially Random Forest—to improve early identification of high-risk pregnancies could inform targeted monitoring and intervention strategies, ultimately contributing to improved maternal cardiovascular health outcomes.

## Limitation of the study

Despite the promising results, this study has several limitations. First, the analysis was based on routinely collected antenatal care data from a single hospital in Bishoftu, Ethiopia, which may limit the external validity and generalizability of the findings to other regions, healthcare settings, or populations with different demographic or clinical characteristics. External validation using independent datasets was not performed and should be considered in future studies to assess the transportability of the proposed models.

Second, although internal validity was strengthened through participant-level data splitting and cross-validation, the relatively limited sample size and the small number of outcome events may still pose a risk of overfitting, particularly for complex machine learning models.

Third, the study focused exclusively on pulse pressure as a surrogate marker of cardiovascular risk, without incorporating other hemodynamic or biochemical indicators that may influence maternal outcomes.

Fourth, the observational nature of the study precludes causal inference, and residual confounding due to unmeasured variables cannot be ruled out.

Finally, potential issues related to missing data, measurement error, and variability in the timing of repeated measurements inherent in longitudinal clinical records were not explicitly modeled and may have influenced the results.

Future research should involve larger, multi-center cohorts, incorporate additional clinically relevant predictors, and include external validation to confirm the robustness and generalizability of machine learning approaches for modeling longitudinal pulse pressure during pregnancy.

## Conclusion

This study demonstrates that machine learning models, particularly the Random Forest approach, provide superior predictive accuracy for modeling longitudinal pulse pressure in pregnant women compared with traditional generalized linear mixed models. Key predictors included maternal weight, age, and recent pulse pressure measurements (lag1 and lag2), underscoring the combined importance of demographic characteristics and temporal dependency in maternal cardiovascular dynamics. Gestational age at admission also emerged as an influential factor, reflecting expected physiological adaptations during pregnancy.

The variable importance analysis further highlighted the dominant role of weight, age, and historical pulse pressure values in explaining variability over time. These findings suggest that machine learning methods can effectively leverage routinely collected antenatal data to capture complex, nonlinear, and time-dependent relationships that may be inadequately represented by conventional statistical models. Consistent with recent ML applications in obstetric risk prediction, our results reinforce the potential of flexible, data-adaptive models to enhance maternal health research.

From a clinical and health system perspective, the application of such models has the potential to support improved maternal cardiovascular monitoring and risk stratification during routine antenatal care. Future research should focus on external validation in diverse clinical settings, integration of additional cardiovascular and behavioral risk factors, and evaluation of real-time implementation within prenatal care workflows to facilitate earlier detection and improved management of hypertensive complications during pregnancy.

## Availability of data and materials

The data that support the findings of this study are available from the corresponding author upon reasonable request.

## CRediT authorship contribution statement

**Merga Abdissa Aga:** Writing – review & editing, Writing – original draft, Visualization, Validation, Supervision, Software, Resources, Project administration, Methodology, Investigation, Funding acquisition, Formal analysis, Data curation, Conceptualization.

## Consent for publication

Not applicable.

## Ethics approval and consent to participate

This study utilized secondary data extracted from hospital follow-up charts to assess maternal survival outcomes. Ethical approval was obtained from the Research Ethical Committee of Bishoftu General Hospital, Bishoftu city, Ethiopia (Approval Number: N/C/S/C/0032/2015 E.C., dated 30/05/2023). Given the retrospective nature of the study, the requirement for informed consent was waived. All patient data were anonymized and handled confidentially to protect participant privacy in accordance with the Declaration of Helsinki and local ethical guidelines.

## Funding statement

No funding was received for this research.

## Declaration of competing interest

The authors declare no conflict of interest related to this research.

## Appendix A Supplementary data

Supplementary Material 1. Hospital-based data extraction tool used to collect clinical and demographic information for predicting pulse pressure among pregnant women. Image 1

## References

[1] WHO (2011).

[2] Say L., Chou D., Gemmill A., Tunçalp Ö., Moller A.-B., Daniels J. (2014). Global causes of maternal death: a WHO systematic analysis. Lancet Glob Health.

[3] Magee L.A., von Dadelszen P., Stones W., Mathai M. (2016).

[4] American College of Obstetricians and Gynecologists (2020). Gestational hypertension and preeclampsia: ACOG practice bulletin, number 222. Obstetr. Gynecol..

[5] Wu P., Haththotuwa R., Kwok C.S., Babu A., Kotronias R.A., Rushton C. (2023). Pre-eclampsia and future cardiovascular health: a systematic review and meta-analysis. Eur J Prev Cardiol.

[6] Franklin S.S., Gustin W., Wong N.D., Larson M.G., Weber M.A., Kannel W.B. (1999). Hemodynamic patterns of age-related changes in blood pressure: the Framingham Heart Study. Circulation.

[7] Safar M.E., London G.M. (2000). Arterial and venous compliance in sustained essential hypertension. Hypertension.

[8] Vlachopoulos C., O’Rourke M., Nichols W.W. (2021).

[9] Blacher J., Asmar R., Djane S., London G.M., Safar M.E. (2000). Aortic pulse wave velocity as a marker of cardiovascular risk in hypertensive patients. Hypertension.

[10] Zhong Q., Hu M., Luo X., He J. (2021). Pulse pressure and cardiovascular disease: an updated review. Clin Hypertens.

[11] Melchiorre K., Sharma R., Thilaganathan B. (2022). Cardiovascular physiology in pregnancy: adaptation and maladaptation in maternal hemodynamics. Ultrasound Obstet Gynecol.

[12] Robson S.C., Hunter S., Boys R.J., Dunlop W. (1989). Serial study of factors influencing changes in cardiac output during human pregnancy. Am J Physiol.

[13] Meah V.L., Cockcroft J.R., Backx K. (2016). Cardiac output and related haemodynamics during pregnancy: a series of meta-analyses. Heart.

[14] Auger N., Fraser W.D., Healy-Profitós J., Arbour L. (2017). Association between preeclampsia and preterm birth by severity and clinical subtype: retrospective cohort study. BMC Pregnancy Childbirth.

[15] Seely E.W., Tsigas E.Z. (2015). Preeclampsia and future cardiovascular disease. Circulation.

[16] Nafisi S., Heidarzadeh M., Doshmangir L. (2021). The relationship between hypertensive disorders of pregnancy and adverse pregnancy outcomes. Preg Hypertens.

[17] Gupta S., Gulati S., Goyal R. (2020). Longitudinal data analysis in clinical research. J Pract Cardiovasc Sci.

[18] Burton G.J., Jauniaux E., Murray A.J. (2022). Oxygen and development of the human placenta. Am J Obstetr Gynecol.

[19] Osungbade K.O., Ige O.K. (2011). Public health perspectives of preeclampsia in developing countries: implication for health system strengthening. J Pregnancy.

[20] Poon L.C., Rolnik D.L., Nicolaides K.H. (2022). Maternal hemodynamics and hypertensive disorders in pregnancy. Prenat Diagn.

[21] Beam A.L., Kohane I.S. (2018). Big data and machine learning in health care. JAMA.

[22] Topol E.J. (2019). High-performance medicine: the convergence of human and artificial intelligence. Nat Med.

[23] Cai X., Chen Y., Wang X., Li J., Chen H. (2023). Machine learning for predicting adverse pregnancy outcomes: a systematic review. Front Cardiovasc Med.

[24] Little R.J.A., Rubin D.B. (2019).

[25] Diggle P.J., Heagerty P., Liang K.Y., Zeger S.L. (2002).

[26] Hastie T., Tibshirani R., Friedman J. (2009).

[27] Kuhn M., Johnson K. (2013).

[28] Laird N.M., Ware J.H. (1982). Random-effects models for longitudinal data. Biometrics.

[29] Pinheiro J.C., Bates D.M. (2000).

[30] McCullagh P., Nelder J.A. (1989).

[31] Breiman L. (2001). Random forests. Mach Learn.

[32] Chen T., Guestrin C. (2016). Proceedings of the 22nd ACM SIGKDD international conference on knowledge discovery and data mining.

[33] Bergstra J., Bengio Y. (2012). Random search for hyper-parameter optimization. J Mach Learn Res.

[34] Kohavi R. (1995). A study of cross-validation and bootstrap for accuracy estimation and model selection. Int Joint Conf Artif Intell.

[35] Smith A.B. (2018). Longitudinal modeling of blood pressure in pregnancy. J Hypertens.

[36] Johnson L., Lee M. (2020). Mixed-effects modeling of cardiovascular data. Stat Methods Med Res.

[37] Goldstein B.A. (2019). Machine learning for health research: prospects and challenges. J Am Med Inform Assoc.

[38] Kwon J.M. (2021). Machine learning for prediction of hypertensive disorders in pregnancy. Comput Biol Med.

[39] Davis A.R. (2017). Using prior blood pressure to predict preeclampsia. Hypertens Pregnancy.

[40] Hernandez G. (2019). Temporal dynamics in blood pressure prediction during pregnancy. BMC Pregnancy Childbirth.

[41] Brown M.A. (2016). Physiological basis of hypertension in pregnancy. Lancet.

[42] Rajkomar A. (2018). Scalable and accurate deep learning for electronic health records. npj Digit Med.

[43] Esteva A. (2019). A guide to deep learning in healthcare. Nat Med.

[44] Miotto R. (2017). Deep learning for healthcare: review, opportunities, and challenges. Brief Bioinform.

[45] Tonekaboni S. (2019). What clinicians want: Explainable AI for healthcare. https://arxiv.org/abs/1905.10226.

[46] Lundberg S.M., Lee S.-I. (2017). Advances in neural information processing systems.

