# Supplementary material for: Machine learning-based identification of determinants of pulse pressure in pregnant women
Source: Glob Epidemiol. 2026 Jan 7;11:100245. doi: 10.1016/j.gloepi.2026.100245 (PMC12818141; doi:10.1016/j.gloepi.2026.100245)
Supplement: Supplementary file 1 — Supplementary Material 1. Hospital-based data extraction tool used to collect clinical and demographic information for predicting pulse pressure among pregnant women. [file mmc1.docx]

**Supplementary Material S1**

**Antenatal Care Data Collection Instrument for Longitudinal Pulse Pressure Study**

**Purpose:** This instrument was used to collect maternal demographic, obstetric, clinical, and longitudinal blood pressure data from antenatal care (ANC) records and follow-up visits. All information was collected repeatedly for each woman across ANC visits.

**Note:** This is a **blank copy** of the questionnaire/data extraction form used during data collection. No participant identifiers are included.

**Section A: Participant Identification**

- Participant Code: __________________________
- Health Facility: ___________________________
- Date of First ANC Visit: ____ / ____ / ______

**Section B: Maternal Demographic Characteristics**

1. **Maternal Age (years):** ________ years
2. **Maternal Weight (kg):** ________ kg

**Section C: Obstetric History**

1. **Gravidity:**
   ☐ Primigravida
   ☐ Multigravida
2. **History of Abortion:**
   ☐ Yes
   ☐ No
3. **History of Preeclampsia (previous pregnancy):**
   ☐ Yes
   ☐ No

**Section D: Current Pregnancy Status**

1. **Gestational Age at Visit (weeks):** ________ weeks
2. **Pregnancy Multiplicity:**
   ☐ Singleton
   ☐ Twin
3. **Number of ANC Visits (up to current visit):**
   ☐ < 4 visits
   ☐ ≥ 4 visits

**Section E: Clinical Characteristics**

1. **Any Comorbidity (e.g., diabetes, chronic hypertension):**
   ☐ Yes
   ☐ No
2. **Complications Developed During Current Pregnancy:**
   ☐ Yes
   ☐ No
3. **Use of Antihypertensive Medication:**
   ☐ Yes
   ☐ No

**Section F: Family History**

1. **Family History of Blood Pressure / Hypertension:**
   ☐ Yes
   ☐ No

**Section G: Longitudinal Blood Pressure Measurements**

*(To be completed at each ANC visit)*

| **Visit Number** | **Visit Date** | **SBP (mmHg)** | **DBP (mmHg)** | **Pulse Pressure** |
| --- | --- | --- | --- | --- |
| Visit 1 | ____ | ______ | ______ | ______ |
| Visit 2 | ____ | ______ | ______ | ______ |
| Visit 3 | ____ | ______ | ______ | ______ |
| Visit 4 | ____ | ______ | ______ | ______ |
| Visit 5 | ____ | ______ | ______ | ______ |
| Visit 6 | ____ | ______ | ______ | ______ |
| Visit 7 | ____ | ______ | ______ | ______ |
| Visit 8 | ____ | ______ | ______ | ______ |
